# Case Report: Gait training using a wearable ankle-exoskeleton in a pediatric patient with Guillain–Barré syndrome

**DOI:** 10.3389/fped.2026.1900528

**Published:** 2026-09-03

**Authors:** David Cordero-Cuevas, Jimena Figueroa-Valero, Elizabeth Pérez-Rodríguez, Raide Alfonso González-Carbonell

**Affiliations:** 1 Association for People with Cerebral Palsy, Mexico City, Mexico; 2 Facultad de Ciencias de la Salud, Universidad Anáhuac Mexico, Mexico City, Mexico; 3 Faculty of Engineering, La Salle University Mexico, Mexico City, Mexico; 4 National School of Higher Studies Juriquilla, National Autonomous University of Mexico, Querétaro, Mexico

**Keywords:** case report, gait analysis, Guillain–Barré syndrome, surface electromyography, wearable ankle-exoskeleton

## Abstract

**Introduction:**

Guillain–Barré syndrome (GBS) is an acute inflammatory demyelinating polyradiculoneuropathy that can lead to severe gait impairment and functional limitations in pediatric patients. Although conventional physiotherapy (CT) is the standard of care, recovery is often prolonged and characterized by persistent deficits in strength and motor control. Wearable ankle-exoskeletons have emerged as a potential adjunctive tool for task-specific gait training; however, their application in pediatric GBS remains largely unexplored. The aim of this single case report was to describe the clinical application of a structured gait training program using the Biomotum SPARK® wearable ankle-exoskeleton (SPARK®) combined with CT in a pediatric patient with GBS.

**Results:**

Discrete variations in muscle strength, spatiotemporal parameters, pelvic kinematics, and muscle activation patterns were recorded between baseline (T0) and post-intervention (T2) assessments. Clinically, manual muscle testing scores suggested localized gains, particularly in bilateral hip abductors (moving from 3/5 to 4/5). Instrumented gait analysis indicated a baseline cadence variation from 101.7 to 108.5 steps/min, alongside a displacement of the gait symmetry index from 1.17% to 0.67%. Pelvic kinematic profiles suggested a trend toward alignment with normative reference bands, notably in pelvic obliquity and right pelvic rotation. Furthermore, surface electromyography recordings suggested localized modifications at T2, characterized by a lower envelope amplitude in the right gastrocnemius medialis activation pattern during the swing-phase (60%–100%) and an increase in amplitude within the normative activation periods of the left tibialis anterior.

**Conclusions:**

This single case report describes the clinical application of an integrated gait training protocol using the SPARK®, which was well-tolerated by the pediatric patient during the recovery-phase of GBS. However, these preliminary observations cannot be generalized or attributed solely to the wearable ankle-exoskeleton, as they likely reflect the combined, synergistic influence of spontaneous neurological recovery, conventional rehabilitation, motor learning, and increased walking exposure. Further controlled studies are necessary to evaluate the potential role and efficacy of wearable ankle-exoskeletons in pediatric GBS rehabilitation.

## 1 Introduction

Guillain–Barré syndrome (GBS) is an acute immune-mediated inflammatory polyradiculoneuropathy of the peripheral nervous system, with an annual global incidence of approximately 1–2 cases per 100,000 person-years, and one of the leading causes of acute flaccid paralysis worldwide (1). It is clinically characterized by rapidly progressive, symmetrical muscle weakness, hyporeflexia or areflexia, and flaccid paralysis with a typically ascending distribution; sensory disturbances and ataxia may also be present depending on the variant and extent of peripheral nerve involvement (2, 3). GBS typically consists of a progressive-phase reaching its nadir within four weeks of symptom onset, followed by a plateau and a recovery phase that may last months or years (2, 3).

Rehabilitation is an essential component of GBS management. During the acute-phase, interventions aim to prevent complications related to immobility, whereas the recovery-phase focuses on restoring strength, balance, and gait through progressive task-oriented training (2, 4). In recent years, robotic-assisted rehabilitation has gained attention as an adjunct to conventional physiotherapy (CT) for neurological gait disorders. Robotic gait systems provide intensive, repetitive, task-specific practice, with the strongest evidence reported in stroke rehabilitation (5, 6). However, their widespread implementation remains limited by cost and accessibility, increasing interest in portable wearable exoskeletons (7).

Wearable ankle-exoskeleton devices have emerged as a promising alternative to address fatigue, residual weakness, and reduced tolerance to intensive rehabilitation (1). These lightweight systems deliver joint-specific assistance or resistance during overground or treadmill walking while promoting active patient participation and task-specific motor learning (8, 9). Evidence from pediatric neurorehabilitation, particularly in populations with cerebral palsy, indicates that wearable ankle-exoskeletons can improve spatiotemporal gait parameters, enhance muscle recruitment, and optimize walking economy (10–13). The Biomotum SPARK® is one of such devices, designed to provide adaptive assistance or resistance to dorsiflexion and plantarflexion in real time during gait (14).

Despite these advancements, there is a lack of published evidence regarding the application of wearable ankle-exoskeletons in pediatric patients with demyelinating peripheral neuropathies such as GBS. To our knowledge, this is among the first published reports describing the clinical application of a wearable ankle-exoskeleton in a pediatric patient with GBS. Therefore, this case report describes the clinical application of a structured gait training program using the SPARK® wearable ankle-exoskeleton combined with CT in a pediatric patient with GBS, contributing preliminary clinical observations to an underexplored area of pediatric neurorehabilitation.

## 2 Case presentation

A 10-year-old female patient with a diagnosis of GBS, established by a pediatric neurologist during the acute-phase, was referred to the Asociación Pro-Personas con Parálisis Cerebral (APAC), Mexico City, approximately four months after disease onset. The diagnosis was established based on the characteristic clinical presentation, electrophysiological evaluation, and complementary imaging studies. Nerve conduction studies and electromyography demonstrated bilateral common peroneal nerve involvement with mixed axonal and demyelinating features, consistent with the diagnosis. During the acute-phase, the patient received standard medical treatment consisting of five consecutive days of intravenous immunoglobulin (IVIG) and paracetamol.

The acute-phase was characterized by lower back pain, progressive loss of dexterity, generalized fatigue, and gait impairment. At the time of referral, pain had resolved; however, persistent fatigue, muscle weakness, and gait dysfunction remained. Neurological examination at admission demonstrated generalized hypotonia affecting both the upper and lower extremities, preserved superficial sensation, decreased lower-limb deep tendon reflexes (patellar and Achilles), preserved upper-limb reflexes, full passive range-of-motion, no pathological reflexes, and no clinical signs of spasticity. Functional gait assessment revealed independent ambulation with an increased base of support, impaired trunk–pelvic dissociation, poor single-leg balance, and difficulty performing tandem gait, heel walking, toe walking, and squatting without assistance. The patient also presented bilateral lower-limb weakness, reduced functional mobility, and limitations in daily-living-activities, particularly stair negotiation and prolonged walking, consistent with residual neuromuscular deficits during the recovery-phase of GBS.

No relevant family history, psychosocial factors, or genetic disorders associated with the patient's condition were reported.

### 2.1 Clinical findings

At baseline (T0), manual muscle testing (Lovett/Kendall scale) demonstrated proximal and distal weakness, with scores ranging from 3/5 to 4/5 across most muscle groups. The greatest deficits were observed in the right lower limb, specifically in the hip flexors, extensors, abductors, adductors, knee flexors, knee extensors, ankle dorsiflexors, and plantar flexors (3/5), while bilateral ankle evertors were also reduced (3/5). Left-sided strength was comparatively better in selected muscle groups, although persistent weakness remained in hip stabilizers and distal ankle musculature (Table 1).

**Table 1:** Clinical muscle strength and spatiotemporal gait outcomes across baseline (T0), mid-intervention (T1), and post-intervention (T2) assessments.

	T0		T1		T2	
	Right-side	Left-side	Right-side	Left-side	Right-side	Left-side
Lovett/Kendall score						
Hip flexors	3	4	4	4	4	4
Hip extensors	3	3	4	4	4	4
Hip abductors	3	3	3	3	4	4
Hip adductors	3	3	3	3	4	4
Knee flexors	3	3	3	4	4	4
Knee extensors	3	4	4	4	4	4
Ankle dorsiflexors	3	4	4	4	4	4
Ankle plantar flexors	3	4	4	4	4	4
Ankle inverters	3	3	4	3	4	4
Gait parameters						
Cadence (steps/min)	101.7 (2.6)		109.5		108.5	
Symmetry index (%)	1.17		1.17		0.67	
Stance-phase (%)	59.2 (1.7)	59.9 (1.6)	60.1 (1.4)	60.8 (3.4)	59.5 (1.9)	59.9 (1.7)
Swing-phase (%)	40.8 (1.7)	40.1 (1.6)	39.9 (1.4)	39.2 (3.4)	40.5 (1.9)	40.1 (1.7)
1st double-support (%)	10.5 (2)	8.7 (0.8)	9.9	9.2 (1.9)	9.5 (0.3)	10.1 (2.3)
Pelvis tilt (°)	11.73 (2.21)	11.53 (1.97)	11.08 (1.6)	10.82 (1.25)	11.7 (1.57)	11.22 (1.83)
Pelvis obliquity (°)	2.17 (1.36)	−1.99 (1.54)	2.68 (1.92)	−2.47 (2.33)	−1.61 (1.57)	1.29 (1.82)
Pelvis rotation (°)	−1.58 (2.33)	1.97 (2.4)	−5.26 (2.48)	5.05 (2.61)	2.33 (1.10)	−3.10 (1.87)

Gait parameters are presented as mean (standard deviation) when available.

Instrumented gait analysis at T0 suggested an altered walking pattern characterized by a baseline cadence of 101.7 (±2.6) steps/min and a gait symmetry index of 1.17%. The initial double-support time was recorded at 10.5% (±2.0%), which sits near typical normative reference values (∼10%). Similarly, the temporal distribution of the gait cycle aligned closely with typical physiological proportions (60/40); the stance-phase occupied approximately 59.2% (±1.7%) of the gait cycle on the right side and 59.9% (±1.6%) on the left, with a corresponding swing-phase duration of 40.8% (±1.7%) and 40.1% (±1.6%), respectively. Consequently, the baseline deviations and altered gait behavior are primarily reflected in the reduced step frequency and discrete inter-limb asymmetry rather than macro-temporal phase distributions.

Pelvic kinematic analysis indicated variations in trunk-pelvic control during walking, with movement profiles shifting outside the normative reference bands. Quantitative deviations at baseline included a mean pelvic tilt of 11.73° (±2.21°) on the right side and 11.53° (±1.97°) on the left. Mean pelvic obliquity presented values of 2.17° (±1.36°) on the right and −1.99° (±1.54°) on the left, while pelvic rotation profiles showed deviations of −1.58° (±2.33°) on the right and 1.97° (±2.40°) on the left. These proximal asymmetries and rotational deviations were more prominent on the right side, which clinically corresponded with the greater deficits in muscle strength identified during manual muscle testing (MMT scores of 3/5 across the right hip extensors, abductors, and adductors). These initial findings suggest the presence of compensatory proximal movement strategies, where altered pelvic kinematics likely served to stabilize or advance the lower limb during the gait cycle to compensate for the greater unilateral muscle weakness and altered balance control.

### 2.2 Timeline of treatment

#### 2.2.1 Therapeutic intervention

Because the patient's primary impairments included persistent lower-limb weakness, gait asymmetry, and reduced walking efficiency despite completion of acute medical treatment, gait training using the Biomotum SPARK® wearable ankle-exoskeleton (SPARK ®) was selected as an adjunct to CT to provide task-specific ankle assistance while preserving active patient participation. Accordingly, the patient was enrolled in a structured neurorehabilitation program incorporating gait training using the SPARK ®. Pharmacological treatment had been completed before referral and remained unchanged throughout rehabilitation. No occupational therapy was provided during the study period.

The SPARK® provides powered assistance during dorsiflexion and plantarflexion while allowing natural walking. The device consisted of instrumented insoles incorporating force-sensitive resistor sensors to detect different phases of the gait cycle, bilateral tibial cuffs secured with Velcro straps, a lumbar-mounted motor unit and battery attached through a magnetic belt, and shoulder straps to distribute the weight of the system.

The SPARK® was operated in its default assistance mode, in which the manufacturer's predefined assistance settings correspond to 15% BW during the stance phase and 3% BW during the swing phase. These settings remained constant throughout the intervention. Based on the patient's body weight entered into the software before each session, the SPARK® automatically calculated the assistance torque delivered during gait. Although assistance parameters can be customized (stance: 0%–35% BW; swing: 0%–15% BW), including independent right and left limb adjustments, the default bilateral settings were maintained because the patient presented bilateral lower-limb weakness secondary to GBS.

**Figure 1:**
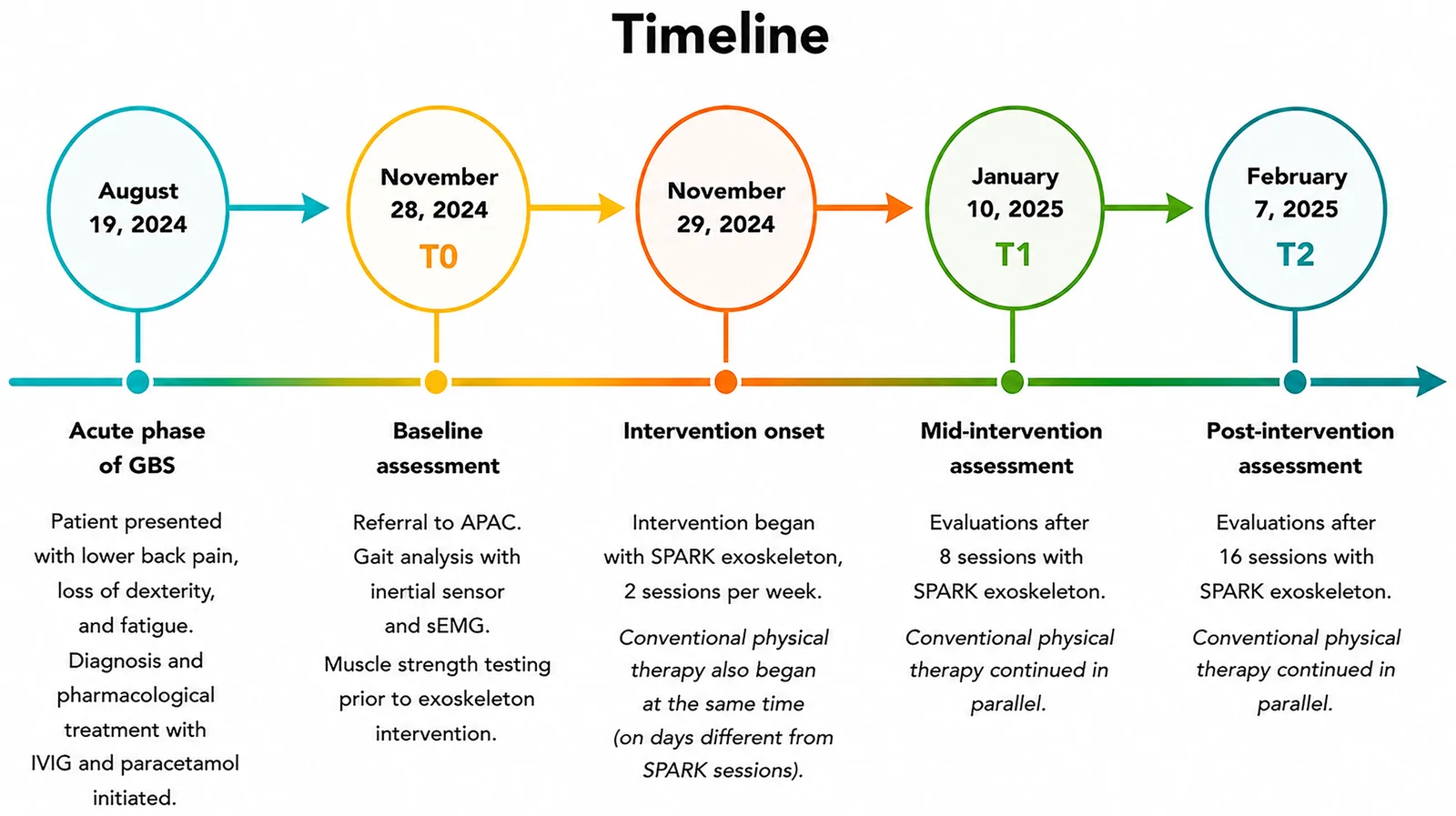
Timeline of the rehabilitation program and clinical assessments. The timeline ranges from the acute-phase of Guillain-Barré Syndrome (GBS) through baseline assessment (T0), mid-intervention assessment after 8 sessions (T1), and post-intervention assessment after 16 sessions (T2). The structured intervention combined robotic gait training using the SPARK wearable ankle-exoskeleton with parallel conventional physical therapy.

The intervention consisted of 16 sessions over eight weeks (two sessions weekly), each lasting approximately 35-minutes (Figure 1). The intervention was performed on a treadmill throughout all 16-sessions, with the device operating in assistive mode. Each session began with placement and adjustment of the exoskeleton, followed by approximately 5-minutes of overground walking to allow familiarization with the device at the patient's self-selected walking speed. The remainder of the session consisted of treadmill-based gait training, performed at an average speed of 1.6 km/h according to the patient's tolerance and perceived exertion.

Progression was achieved by increasing treadmill inclination after the mid-intervention (T1) assessment (Session 8), while exoskeleton assistance remained unchanged. Inclination was increased whenever the patient maintained an appropriate gait pattern without excessive muscular fatigue. Patient-reported qualitative indicators, including perceived fatigue, walking confidence, and treadmill tolerance, were monitored through direct verbal feedback and clinical observation during each session. If fatigue was reported during a session, treadmill walking was temporarily paused for a short rest interval before resuming training once symptoms improved. No sessions were terminated prematurely due to fatigue or adverse events.

All sessions were supervised by a physical therapist, who assisted with donning and doffing the exoskeleton and continuously monitored patient safety, gait quality, and treatment tolerance. During the first four sessions, verbal and manual cues facilitated postural alignment and familiarization. No falls, skin irritation, musculoskeletal injuries, or device-related complications occurred.

In addition to gait training using the SPARK®, the patient received two 45-minutes CT sessions per week throughout the intervention period. CT focused on lower-limb strengthening, core stability, proprioceptive training, gait re-education, pelvic stability, upper-limb strengthening, neuromuscular electrical stimulation of the intrinsic foot muscles, and functional exercises targeting ankle control and postural stability. A home exercise program consisting of exercises similar to those performed during conventional physiotherapy was also prescribed to encourage continued practice between treatment sessions.

### 2.3 Outcome measures

Clinical and instrumented evaluations were performed at three specific time points to assess the patient's evolution: baseline (T0, pre-intervention), mid-intervention (T1, after Session 8), and post-intervention (T2, after Session 16) (Figure 1). Clinician-assessed outcomes included manual muscle testing (MMT) using the Lovett/Kendall scale for bilateral lower-limb muscle groups, comprising the hip flexors, extensors, abductors and adductors; knee flexors and extensors; ankle dorsiflexors, plantarflexors, invertors, and evertors.

Instrumented gait performance was evaluated over a 10-meter path, with the patient completing 15 gait cycles per assessment. Kinematics, spatiotemporal data, and surface electromyography (sEMG) were recorded simultaneously through the FREELAB system (BTS Bioengineering, Milan, Italy). This platform provides native hardware synchronization with all data acquisition and processing managed within the EMG-Analyzer software suite (BTS Bioengineering).

Pelvic kinematic parameters and spatiotemporal gait metrics were calculated directly from the raw data captured by a single inertial sensor (Baiobit, BTS Bioengineering) containing a triaxial accelerometer, gyroscope, and magnetometer, which was secured at the S2 vertebra using an elastic belt to capture data at 100 Hz. Pelvic kinematics profiles were derived from gyroscope data, and the mean and standard deviation of pelvis tilt, rotation and obliquity were computed for the entire gait cycle. Gait events (initial contact, IC and toe-off, TO) were identified from anteroposterior acceleration curves. The temporal durations of the stance, swing, and double-support phases were subsequently extracted and time-normalized to 100% of the gait cycle. Then, the symmetry index was computed as the difference in stance-phase duration between sides (Equation 1), where values close to 0 indicate symmetry.

$$SI=\frac{Stance_{left}-Stance_{right}}{0.5\times (Stance_{left}+Stance_{right})}$$ (1)

Bilateral muscle activation patterns of the tibialis anterior and medial gastrocnemius were assessed via surface electromyography (FREEEMG, BTS Bioengineering). Skin preparation and Ag/AgCl bipolar pre-gelled electrode placement strictly followed SENIAM guidelines. Raw EMG signals were band-pass filtered using a zero-phase fourth-order Butterworth filter (cutoff frequency: 20–400 Hz) to remove movement artifacts and high-frequency noise. The filtered signals were full-wave rectified, and a root-mean-square (RMS) envelope was calculated using a 50-ms moving window. Finally, RMS amplitudes were time-normalized to the gait cycle and averaged across trials.

Patient-assessed outcomes included perceived fatigue during training sessions, walking confidence, and tolerance to treadmill ambulation with the exoskeleton. These outcomes were monitored through direct verbal feedback during each session and therapist observation.

## 3 Discussion

This case report describes the clinical application of gait training using the Biomotum SPARK® wearable ankle-exoskeleton (SPARK®) in a pediatric patient during the recovery-phase of GBS with persistent gait dysfunction. Following the 16-session rehabilitation program, noticeable variations were recorded in lower-limb muscle strength, spatiotemporal gait parameters, pelvic kinematic profiles and muscular activation patterns. These preliminary observations for a single case align with previous reports suggesting that robotic and exoskeleton-assisted gait interventions can potentially support motor recovery, postural control, gait symmetry, and walking efficiency in neurological populations through repetitive, task-specific practice and augmented sensorimotor feedback (7, 15–17). Furthermore, the protocol appeared feasible and well-tolerated in this instance, as reflected by completion of the 16 scheduled sessions over 8 weeks with no missed sessions or documented adverse events, such as falls, musculoskeletal injuries, skin complications, or device-related incidents.

An important clinical finding was the recorded variation in MMT scores across both proximal and distal muscle groups. Although the device primarily assists ankle function, bilateral hip abductor strength improved from 3/5 to 4/5, accompanied by gains in several hip and knee muscle groups (Table 1). These proximal improvements may reflect more efficient gait mechanics and potentially reduced compensatory stabilization demands during walking. Repetitive robotic gait training might support muscle activation adaptations beyond the directly assisted joint (16, 17). In individuals with distal weakness, proximal musculature frequently compensates to maintain balance and limb advancement during gait. By enhancing ankle propulsion and foot clearance, the exoskeleton may have contributed to more efficient lower-limb biomechanics and attenuated compensatory movement profiles, consistent with prior descriptions of propulsion-related adaptations during gait (18).

**Figure 2:**
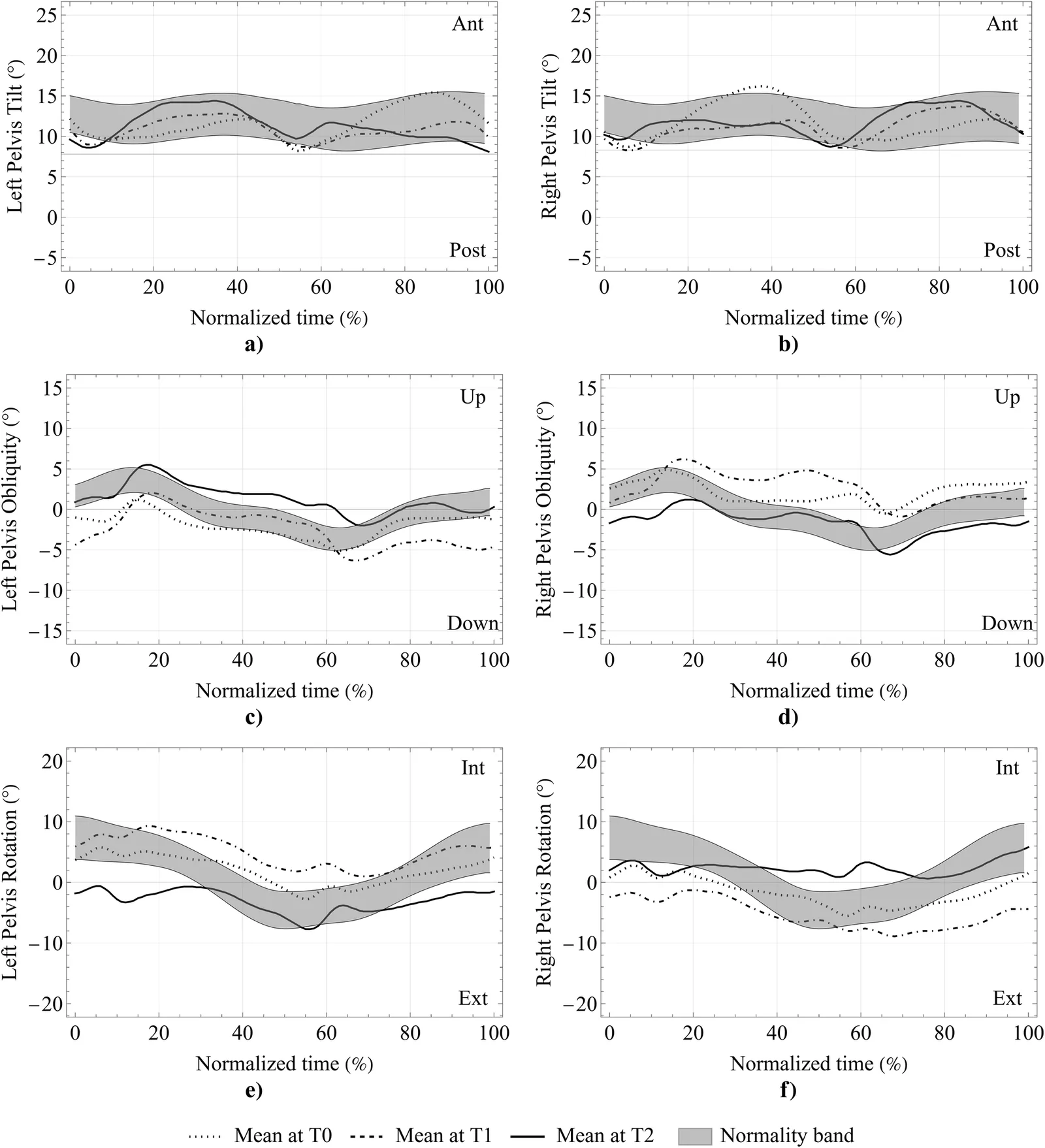
Pelvic kinematic profiles during the normalized gait cycle at baseline (T0; dotted line), mid-intervention (T1; dashed line), and post-intervention (T2; solid line): pelvic tilt **(a,b)**, obliquity **(c,d)**, and rotation **(e,f)** for left **(a,c,e)** and right **(b,d,f)** sides. Gray-shaded areas represent normative bands. Pelvic tilt remained largely within normative ranges, while pelvic obliquity shifted toward normalcy at T2. On the right side **(f)**, which initially exhibited greater clinical impairment, the pelvic rotation profile displaced from negative external ranges at baseline (T0) toward the neutral line and positive internal values at T2; this upward shift away from retraction suggests a change toward pelvic advancement, potentially reflecting a trend of functional recovery during locomotion.

This interpretation is supported by the pelvic kinematic profiles, which suggest a trend toward reduced asymmetry and smoother movement profiles at T2 compared with baseline. The more physiological pelvic tilt, obliquity and rotation may indicate improved frontal and transverse plane control during walking, suggesting decreased compensatory trunk–pelvic motion (Figure 2). Previous gait analyses in patients with peripheral weakness and neurological disorders have shown that enhanced distal control can positively influence proximal kinematics and overall gait symmetry (19, 20). Robotic gait interventions have also been associated with improvements in postural control and gait coordination during locomotion (5, 17). This stabilization of proximal kinematics was clinically mirrored by a qualitative increase in the patient's walking confidence and a progressive tolerance to treadmill ambulation over the course of the sessions, illustrating how objective mechanical support may relate to perceived functional security.

**Figure 3:**
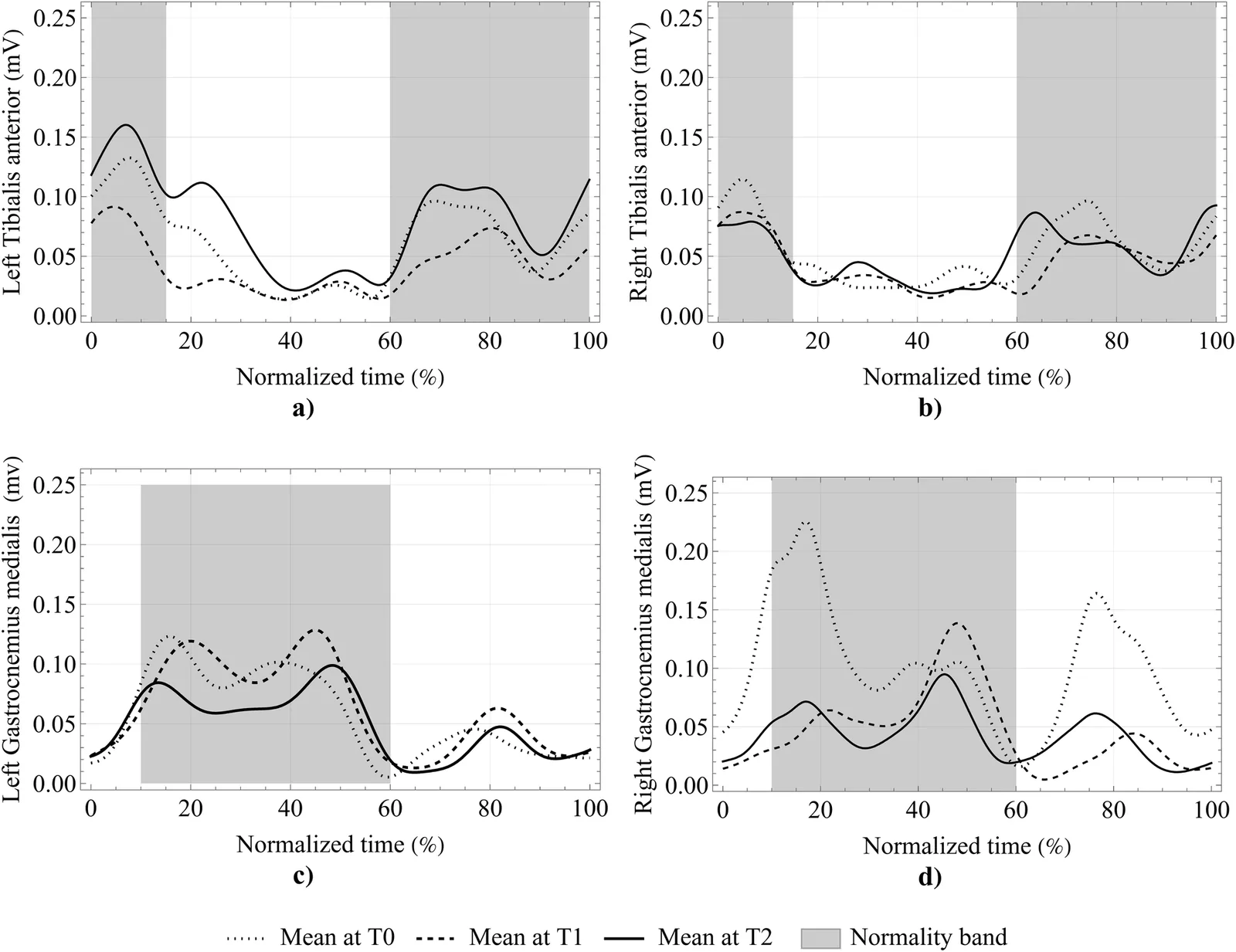
Surface electromyographic (sEMG) activation patterns of the bilateral tibialis anterior **(a,b)** and medial gastrocnemius **(c,d)** muscles normalized to the gait cycle for left **(a,c)** and right **(b,d)** sides. Curves illustrate the mean sEMG envelope (mV) at baseline (T0; dotted line), mid-intervention (T1; dashed line), and post-intervention (T2; solid line), with gray-shaded areas representing normative reference bands of typical activity. On the right side, the gastrocnemius medialis activation pattern displayed a lower amplitude across the gait cycle at T2 compared with baseline (T0), particularly during the swing-phase (60%–100%). Concurrently, the left tibialis anterior activation pattern exhibited an increase in amplitude within its normative periods at T2, suggesting variations in muscle recruitment strategies over the course of the intervention.

Surface electromyography also revealed discrete variations in muscle activation patterns following the intervention (Figure 3). At T2, visual inspection of the recordings suggested modifications in the activation pattern compared with baseline. The right gastrocnemius medialis pattern displayed a lower amplitude across the gait cycle, particularly during the swing-phase, while the left tibialis anterior pattern exhibited an increase in amplitude within its normative activation periods. Comparable electromyographic variations have been described after robotic gait training, including changes in muscle activation timing and reductions in pathological co-contraction patterns (17, 21). These observations align with an adjusted muscle coordination and potentially more efficient motor control strategies during walking. Clinically, although muscular fatigue did occasionally occur during training, it was managed with short rest intervals that allowed the patient to resume ambulation without overtraining, suggesting that the protocol effectively respected the physiological thresholds necessary to consolidate these peripheral motor adaptations.

Spatiotemporal parameters, including increased cadence and a reduced symmetry index, further suggest a trend toward enhanced walking efficiency and gait consistency in this instance. Previous studies evaluating wearable exoskeletons and robotic gait devices in pediatric neurological rehabilitation, particularly in children with cerebral palsy, have reported similar variations in cadence, gait symmetry, and walking performance following repetitive task-oriented gait training (5, 7, 15). Although cerebral palsy differs substantially from GBS, these studies suggest that robotic gait training may facilitate repetitive, task-specific locomotor practice across pediatric neurological conditions. Nevertheless, evidence specifically evaluating wearable ankle-exoskeletons in pediatric GBS remains extremely limited; therefore, comparisons should be interpreted cautiously, and the present findings should be considered preliminary clinical observations.

Several limitations should be considered when interpreting this case report. First, the intervention was initiated approximately four months after disease onset, during the recovery-phase of GBS, when spontaneous neurological recovery is expected to occur (2, 22). Therefore, the observed changes cannot be attributed solely to the gait training using the SPARK® and should instead be interpreted as reflecting the combined influence of the natural recovery process, repeated task-specific gait practice, motor learning, and the use of the wearable ankle-exoskeleton during gait training. In addition, this report describes a single patient without a control condition, which precludes any causal inference regarding treatment effects. Follow-up was limited to the 8-week intervention period because no additional clinical or instrumented assessments were performed after the 16-session rehabilitation program. Consequently, the persistence of the observed changes over time could not be determined. Finally, the MMT was complemented by instrumented gait analysis to provide a broader characterization of gait biomechanics and the muscular function, standardized functional outcome measures, such as walking endurance, balance, participation, fatigue, and patient-reported outcomes, were not collected. Therefore, no conclusions regarding treatment efficacy can be drawn from a single uncontrolled case, and these preliminary observations serve primarily to inform future research. Further controlled studies with larger sample sizes are warranted to evaluate efficacy, determine optimal intervention timing, and establish precise patient selection criteria for interventions using wearable ankle-exoskeletons in pediatric peripheral neuropathies such as GBS. Future prospective investigations should incorporate long-term follow-up, standardized functional and participation measures, lower-limb anthropometric measurements, and ankle and knee kinematics to provide a more comprehensive biomechanical characterization of gait adaptations following robotic rehabilitation.

## 4 Patient perspective

The patient's legal guardian reported that, at the beginning of the rehabilitation program, the patient lacked confidence in her movements, feared fatigue, and showed limited participation in physical activities. Throughout the intervention, she progressively gained confidence in walking and movement, which increased her motivation to continue therapy. The patient completed all scheduled sessions without adverse events. According to her family, improvements were observed in walking stability, endurance, independence in daily activities, and stair negotiation. Her teachers also reported greater participation in classroom and physical education activities. The patient's mother considered the rehabilitation program a positive experience that improved her daughter's confidence, functional independence, and participation in everyday activities.

## Data Availability

The original contributions presented in the study are included in the article/Supplementary Material, further inquiries can be directed to the corresponding author.
